# Identification of dissociation factors in pancreatic Cancer using a mass spectrometry-based proteomic approach

**DOI:** 10.1186/s12885-020-6522-3

**Published:** 2020-01-20

**Authors:** Peng Liu, Lingming Kong, Keke Liang, Yunhao Wu, Haoyi Jin, Bing Song, Xiaodong Tan

**Affiliations:** 1 Department of General Surgery, Shengjing Hospital of China Medical University, Shenyang, 110004 China; 2 Cardiff Institute of Tissue Engineering and Repair, School of Dentistry, Cardiff University, Cardiff, CF14 4XY UK

**Keywords:** Pancreatic cancer, Secreted proteome, SEC, Mass spectrum

## Abstract

**Backgroud:**

Pancreatic cancer is a highly malignant tumor of the digestive system. This secretome of pancreatic cancer is key to its progression and metastasis. But different methods of protein extraction affect the final results. In other words, the real secretion of proteins in cancer cells has been changed. Based on mass spectrometry, we analyze the secretome from the serum-containing and serum-free medium, using different protein pretreatment methods. This study aims to identify dissociation factors in pancreatic cancer.

**Methods:**

In this study, pancreatic cancer cells were cultured in serum-containing or serum-free medium, and the corresponding supernatants were extracted as samples. Subsequently, the above samples were separated by size exclusion chromatography (SEC), and peptide segments were identified by LC-MS/MS. The final results were identified via the hamster secreted protein database and a public database.

**Results:**

Although the number of identified proteins in the serum-free medium group was high, the real secretion of proteins in pancreatic cancer cells was changed. There were six significant secreted proteins in the serum-containing medium group. Survival analysis via the TCGA database suggested that patients with higher expression levels of YWHAG showed a worse overall survival rate than those with lower YWHAG expression.

**Conclusions:**

Our study demonstrated the results in the serum-containing medium group were more similar to the real secretome of pancreatic cancer cells. YWHAG could be used as a prognostic indicator for pancreatic cancer.

## Background

Pancreatic cancer is the fourth leading cause of cancer death worldwide and is characterized by rapid progression, high invasiveness, and resistance to chemotherapeutic drugs. The latest survey of malignant tumors in China showed that the mortality rate of pancreatic cancer ranks sixth [1–3]. More than 80% of patients with pancreatic cancer are diagnosed with local invasion or even distant metastasis. Theoretically, the possibility of surgical resection is lost, and only palliative treatment is tolerated [4]. In addition, patients undergoing radical surgery have a median survival time of only 18 months [5]. Early diagnosis and appropriate treatment can significantly improve the prognosis of pancreatic cancer. With the development of experimental techniques, the number of molecular detection methods for cancer is increasing. These methods play an important role in the early diagnosis of pancreatic cancer [6]. In a previous study, we used two cell lines derived from the hamster model of pancreatic cancer that have distinct invasion and metastasis abilities: a nondissociated, low-metastasis pancreatic cancer cell line (PC-1) and a dissociated, high-metastasis pancreatic cancer cell line (PC-1.0). Conditioned medium was prepared from the purified supernatant of PC-1.0 cells and used to culture PC-1 cells. The growth state of PC-1 cells was changed and exhibited the growth state of PC-1.0 cells. Therefore, we concluded that the supernatant of PC-1.0 cells contains key factors that can promote changes in cell biological behavior, which we call dissociation factors (DF) [7, 8]. The purpose of this experiment was to identify dissociation factors by using different sample pretreatment methods combined with size exclusion chromatography.

## Methods

### Cell lines and cell culture

PC-1 cells grew as islet-like colonies of cells, whereas PC-1.0 cells grew as single cells. The source and incubation conditions of the cells were described previously [9].

### Materials

Acetonitrile (ACN) and methanol were purchased from Merck Company (Germany); glacial acetic acid, from Damao Chemical Reagent Factory in Tianjin; and bovine serum albumin (BSA), from Sigma-Aldrich Company (USA). Trypsin (bovine pancreas), formic acid, trifluoroacetic acid, urea, protease inhibitor cocktail, dithiothreitol, trichloroacetic acid, acetone, and iodoacetamide were purchased from Sigma–Aldrich (St. Louis, MO, USA). All experimental water was purified by a Milli-Q system (Millipore Corporation, USA). A Thermo SEC120 HPLC column (5 μm, 120 Å) was used. An Ultimate 3000 chromatograph and Thermo LTQ-Orbitrap mass spectrometer were used for detection.

### Effects of serum-free conditioned medium from PC-1.0 cells on the activity of PC-1 cells

Preparation of serum-free conditioned medium: Three methods were used to prepare conditioned medium from PC-1.0 cells, which was used to treat cultured PC-1 cells for 24 h; then, morphological changes in PC-1 cells were observed. The following methods were used: Method 1: PC-1.0 cells were washed 5 times with PBS; Method 2: PC-1.0 cells were washed 3 times with PBS and incubated 2 times with phenol-free medium (Gibco, Grand Island, NY) for 20 min each; And Method 3: PC-1.0 cells were incubated in 2% PBS + phenol-free medium for 20 min and in phenol-free medium 4 times for 20 min each. The supernatants of the above samples were extracted and used to prepare the culture medium.

### Extraction of total protein from samples

PC-1.0 cell and PC-1 cell supernatants and RPMI 1640 medium (negative control group) were extracted as samples 4, 5 and 6 in the serum-containing medium experimental group. Each sample was spun at 12000 r/min through a 0.22 μm fiber filter and concentrated using a 3 kDa concentrating tube by centrifuging at 3500×g for 120 min. The protein concentration was measured using the BCA method.

### SEC-RPLC-MS/MS analysis

Low-abundance proteins were enriched on a size exclusion chromatography (SEC) column. The 200 μl sample was washed for 10 min with buffer A at a flow rate of 0.5 ml/min. After collecting the effluent components, the remaining fractions were eluted with buffer B at a flow rate of 1 ml/min for 7 min, and the collected fractions were stored at − 20 °C for use. The effluent components collected were centralized in a rotary concentrator with a 5 kDa molecular weight cutoff membrane and centrifuged at 10 °C for 5000 r/min. Samples were collected for subsequent application.

An Ultimate 3000 chromatograph and a Thermo LTQ-Orbitrap mass spectrometer were used for detection. Peptides were loaded on an in-house-packed C18 capillary trap column (150 μm i.d. × 4 cm) and separated using a C18 separation column (75 μm i.d. × 15 cm). Phase A was 98% H_2_O + 2% ACN with 0.1% FA, and phase B was 2% H_2_O + 98% ACN with 0.1% FA. The gradient was as follows: 0–6% phase B for 10 min, 6–35% phase B for 100 min, 35–80% phase B for 10 min, and 80% phase B for 10 min. The temperature of the ion transfer capillary was set at 275°Cwith a spray voltage of 2.7 kV. The scan range was set from m/z = 300–1800.There was a 20 s exclusion window. Raw spectrum data were searched using Mascot (2.3.2). For classifying the obtained protein results, the database species used in the experiment were both hamster and bovine. Mass tolerances were set at 7 ppm for parent ions and 20 ppm for fragments. The fixed modification was cysteine alkylation, and the variable modification was methionine oxidation. The maximum number of missing cleavage sites was 2, and the FDR was controlled at less than 1%.

### Bioinformatic analysis

Because special structural characteristics of secreted proteins usually include a signal peptide, SignalP4.1 software was used to search the current hamster protein database and to construct the hamster secreted protein database (http://www.cbs.dtu.dk/services/SignalP/, probability > 0.90) [10]. In the serum-containing medium group, RPMI 1640 medium was used as the negative control to eliminate the error caused by unlabeled samples. The results were screened from the database of hamster secretory proteins. Subsequently, the DAVID (http://david.abcc.ncifcrf.gov/) [11] and STING (https://string-db.org/) [12] bioinformatics software tools were used to analyze protein functions and possible interacting proteins. Finally, Survival analysis of patients with the different DFs was analyzed with Kaplan Meier Plotter (http://kmplot.com/analysis/index.php?p=service&cancer=pancancer_rnaseq) [13]. The Gene Expression Profiling Interactive Analysis (GEPIA) database was used to analyze the expression of the target genes in the TCGA database (http://gepia.cancer-pku.cn/) [14]. The expression level of YWHAG in different cancer stages was analyzed with the online analysis platform UALCAN (http://ualcan.path.uab.edu/index.html) [15].

### YWHAG uptake assay

Western blotting was performed as described previously [9]. Primary antibodies against YWHAG and β-actin (Abcam, USA) were used. Samples with equivalent amounts of total protein (20 μg) were loaded. Western blot signals were quantified using an Amersham Imager 600 (GE Healthcare, Little Chalfont, UK), and band signals were expressed as relative protein amounts compared to β-actin. The purified supernatant from PC-1.0 cells were added to PC-1 cells at 60–70% confluence. The YWHAG protein level of the PC-1 cells was tested by western blot analysis after another 24 h of culture. Human pancreatic cancer cell lines AsPC-1 and Capan-2, which have morphological and functional characteristics similar to PC-1.0 and PC-1 cells, respectively, were used to determine if the results from hamster cells coincide with human pancreatic cancer cell lines.

### Statistical analysis

Statistical analyses were conducted and graphics were generated using GraphPad Prism 6.0. *P* < 0.05 was considered statistically significant in this study. Comparisons of quantitative data were made using Student’s t-test.

## Results

### Biological functional validation of serum-free culture conditions

**Fig. 1:**
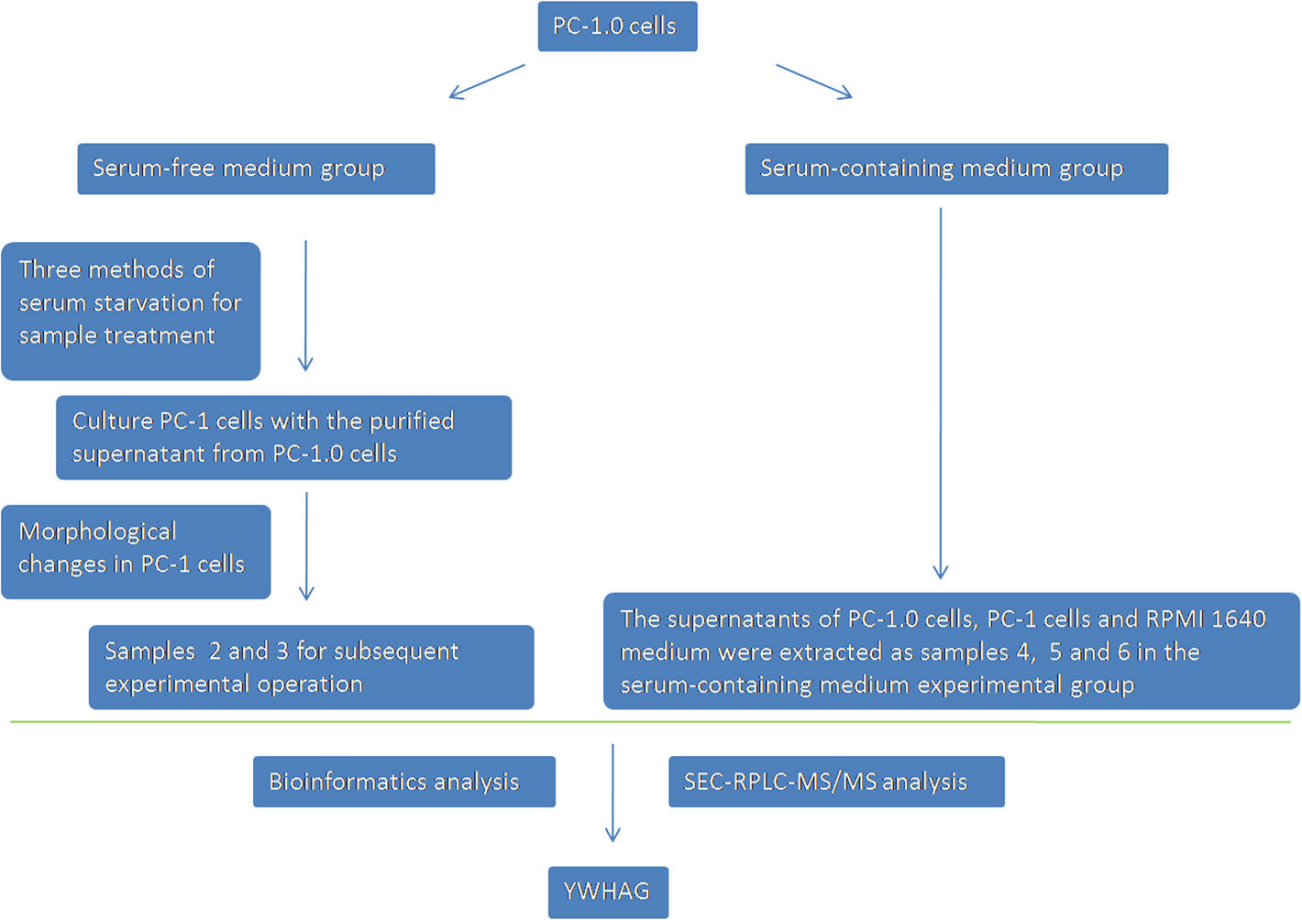
Flowchart of sample procedures used in this study. Serum-free medium group were divided three group using different treated serum starvation. For Sample 1, a large number PC-1 cells died after incubation with conditioned medium from Sample 1. The results show that the serum starvation affect the condition, maybe undergo apoptosis, so the subsequent experiment exclude the Sample1

**Fig. 2:**
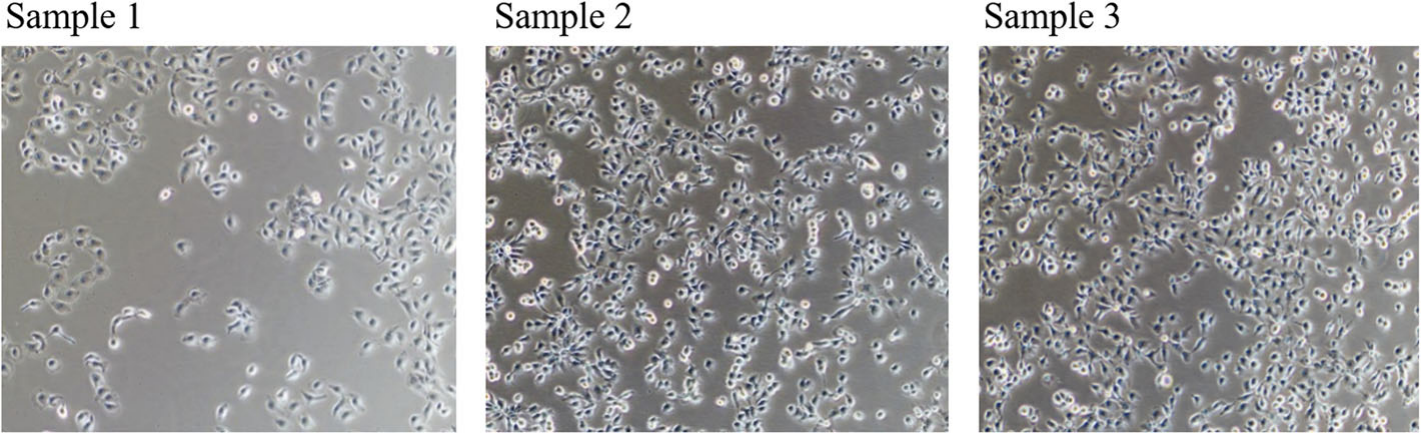
Changes in PC-1.0 cells after serum starvation observed by microscopy

**Fig. 3:**
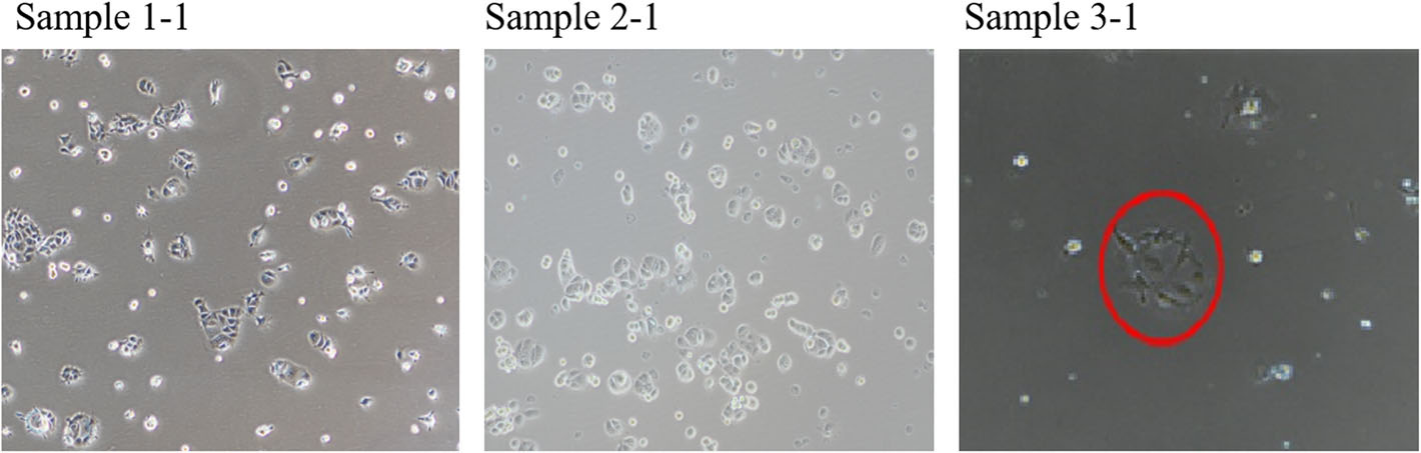
PC-1 cell morphology 12 h after the addition of PC-1.0 supernatant. A large number of PC-1 cells died after incubation with conditioned medium from Sample 1; the number of PC-1 cells after incubation with Sample 2 medium exhibited a relative decrease, and there was no obvious separation trend; and the growth status of PC-1 cells after treatment with Sample 3 medium changed.

According to the purpose of this study, samples of highly invasive PC-1.0 cells were processed and divided into the serum-free and serum-containing groups (Fig. 1). In the serum-free group, PC-1.0 cells were treated with three different serum starvation methods, and the cell content of sample 1 was the lowest (Fig. 2). Subsequently, we extracted the corresponding supernatant to prepare conditioned medium and incubated PC-1 cells with this medium. It was found that a large number of PC-1 cells died after incubation with conditioned medium from Sample 1; the number of PC-1 cells after incubation with Sample 2 medium exhibited a relative decrease, and there was no obvious separation trend; and the growth status of PC-1 cells after treatment with Sample 3 medium changed (Fig.3). Therefore, the serum-free treatment process changed the composition of the original supernatant, which may lead to errors in the final analysis results.

### Identification of DFs

**Fig. 4:**
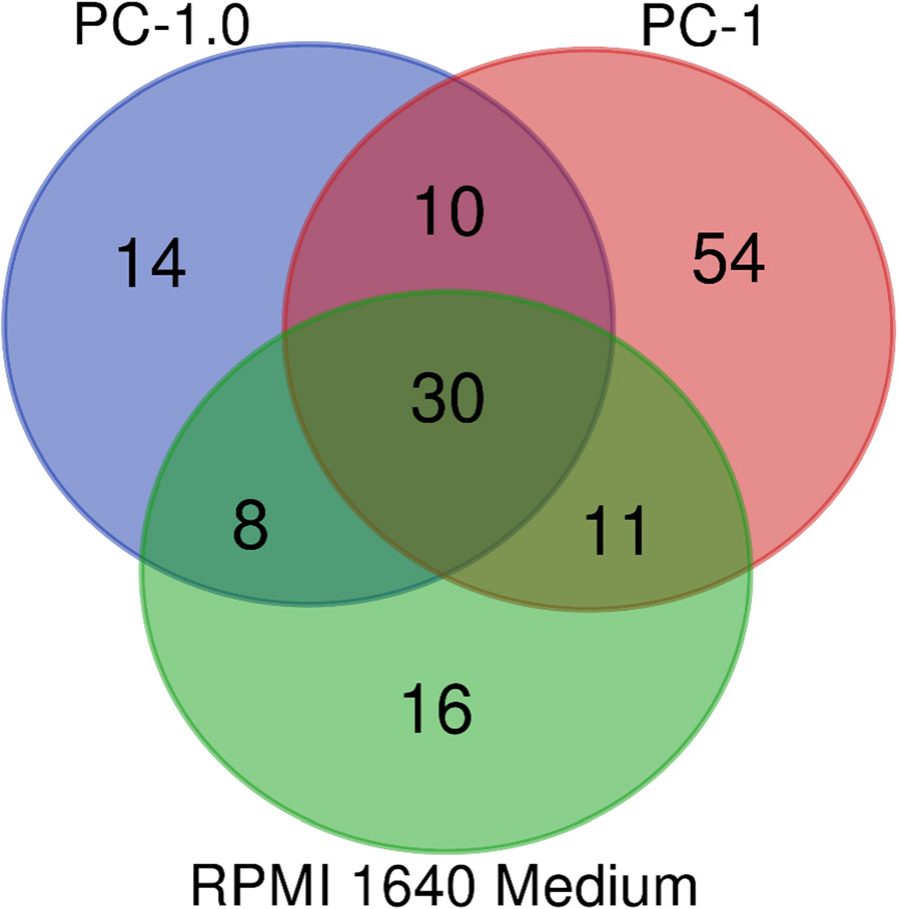
The results of MS in the serum-containing group were shown by a Venn diagram. As a result, 14 protein only exists in the supernatant of PC-1.0 cell lines. Among them, 6 protein, which contain values, may be as candidate DFs

According to the biological function verification results, samples 2 and 3 were subjected to subsequent mass spectrometry verification (Additional file 1). From the serum-containing medium experimental group, samples 4, 5 and 6 were taken for follow-up experiments. Each group was separated by SEC and analyzed by mass spectrometry. The results were searched by Mascot software. The database species used were hamsters and cattle. The results were then searched to generate the database of hamster secreted proteins (see Table 1, Additional files 2 and 3). The results showed that the number of secreted proteins in samples 4, 5 and 6 was small, which indicated that the serum had a great influence on the number of final identified proteins, but the results were more authentic and more conducive to further verification than those from the serum-free groups. The data from the serum-containing experimental groups were integrated and analyzed. Only 6 secretory proteins were expressed in PC-1.0cells: matrix metalloelastase 12 (MMP12), matrix metalloproteinase 1 (stromelysin-2, MMP10), laminin subunit alpha-5 (LAMA5), Tyr-3/Trp-5 monooxygenase activator protein gamma (14–3-3 eta, YWHAG), carboxypeptidase N catalytic chain (CPN1) and coagulation factor V (THPH2) (Fig. 4).

**Table 1:** Protein spectrum data results

Species Sample	Protein	Hamster	Hamster - secreted protein	Bovine
No. 2	1378	987	99	391
No. 3	1496	1092	111	404
No. 4	230	62	25	168
No. 5	284	105	28	179
No. 6	236	65	21	171

### Identification of YWHAG as a prognostic biomarker of pancreatic cancer

**Fig. 5:**
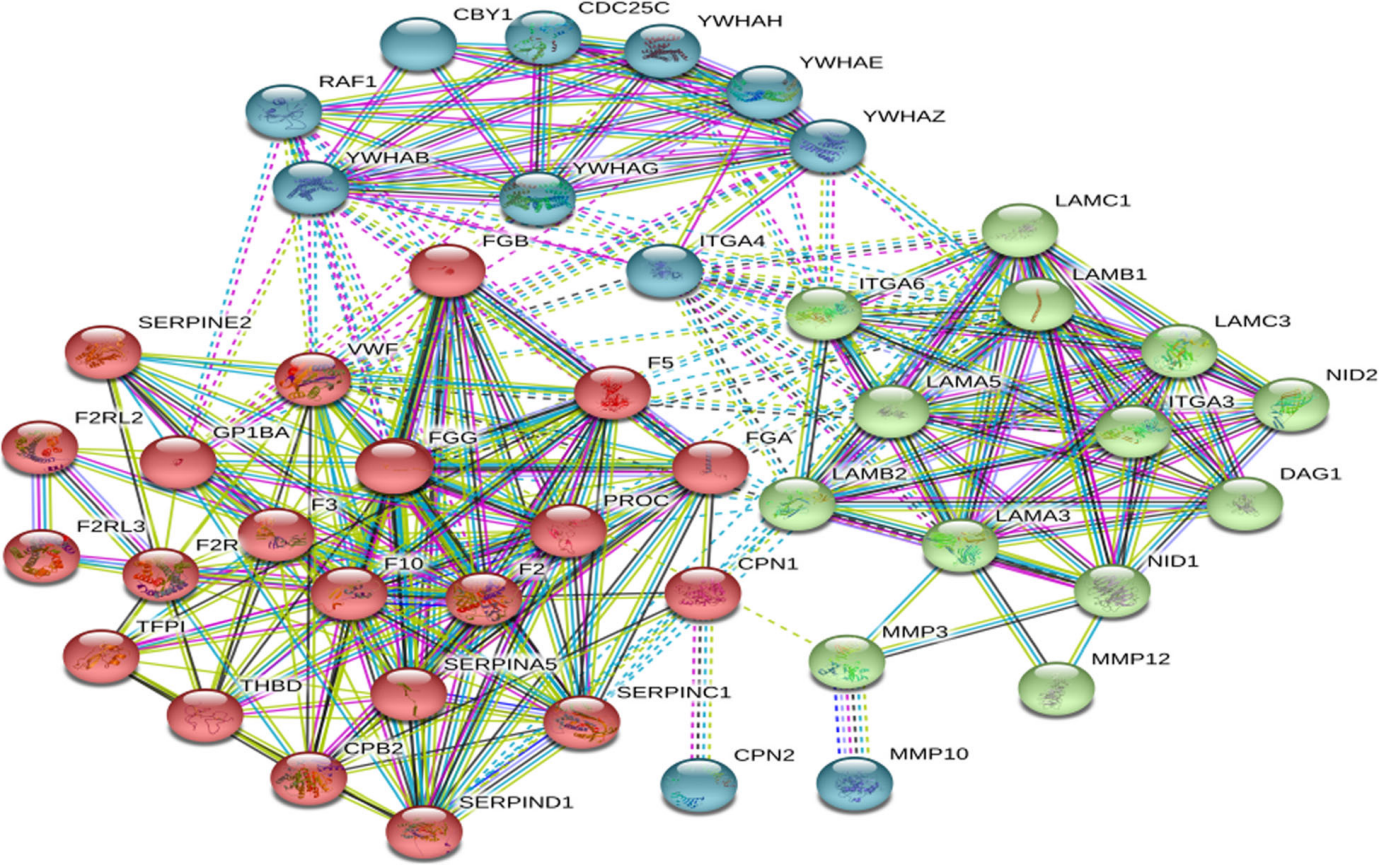
STRING software predicts protein-protein interactions. To further analyze the interaction between the identified proteins, we used the STRING database to retrieve the six proteins.(YWHAG, MMP12, MMP10, LAMA5, CPN1 and F5)

**Fig. 6:**
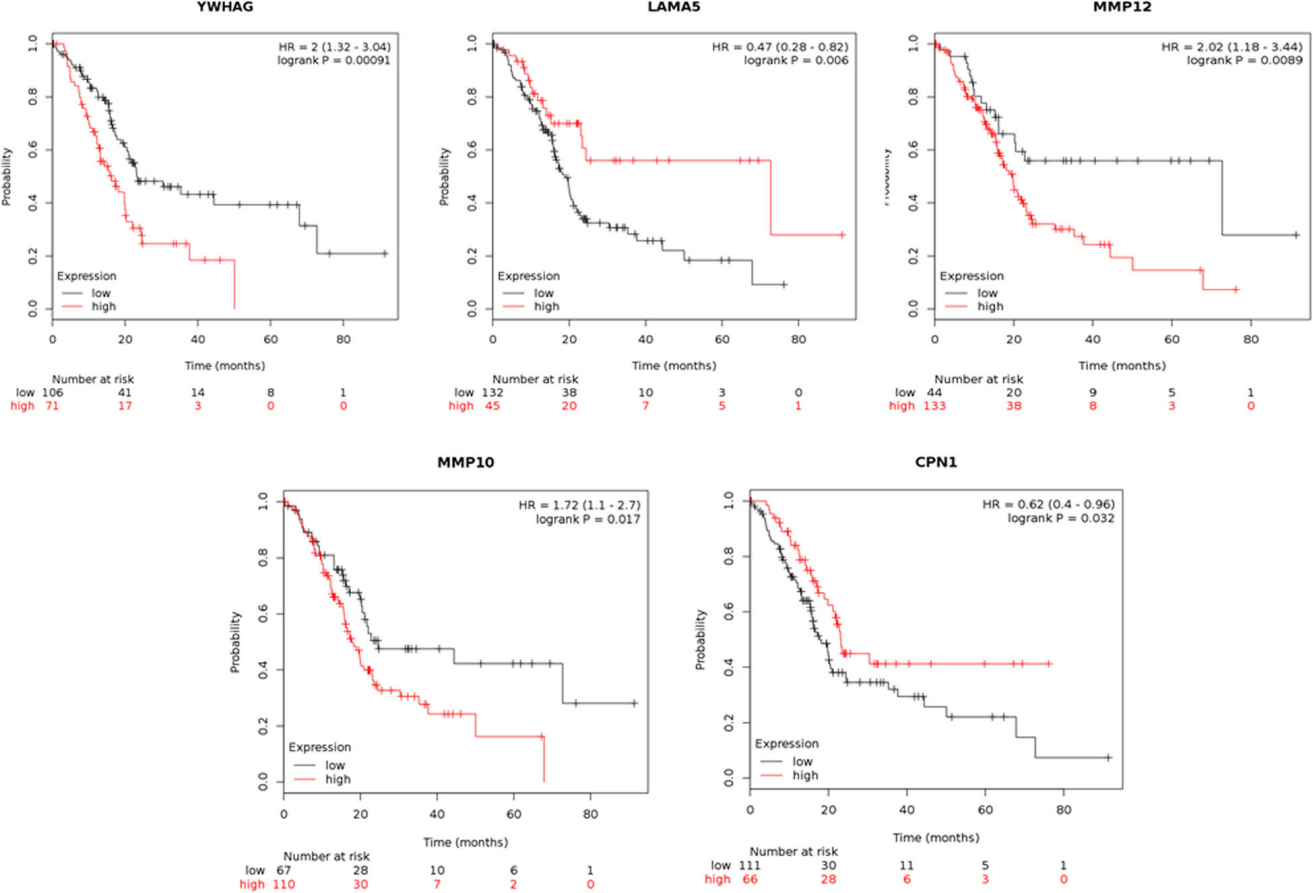
Survival analysis of patients with 5 DFs in the TCGA database. YWHAG can be used as a prognostic indicator for pancreatic cancer. (*P* = 0.00091, FDR < 10%). The FDR of MMP12 and LAMA5 is over 50%. F5 were detected in database

**Fig. 7:**
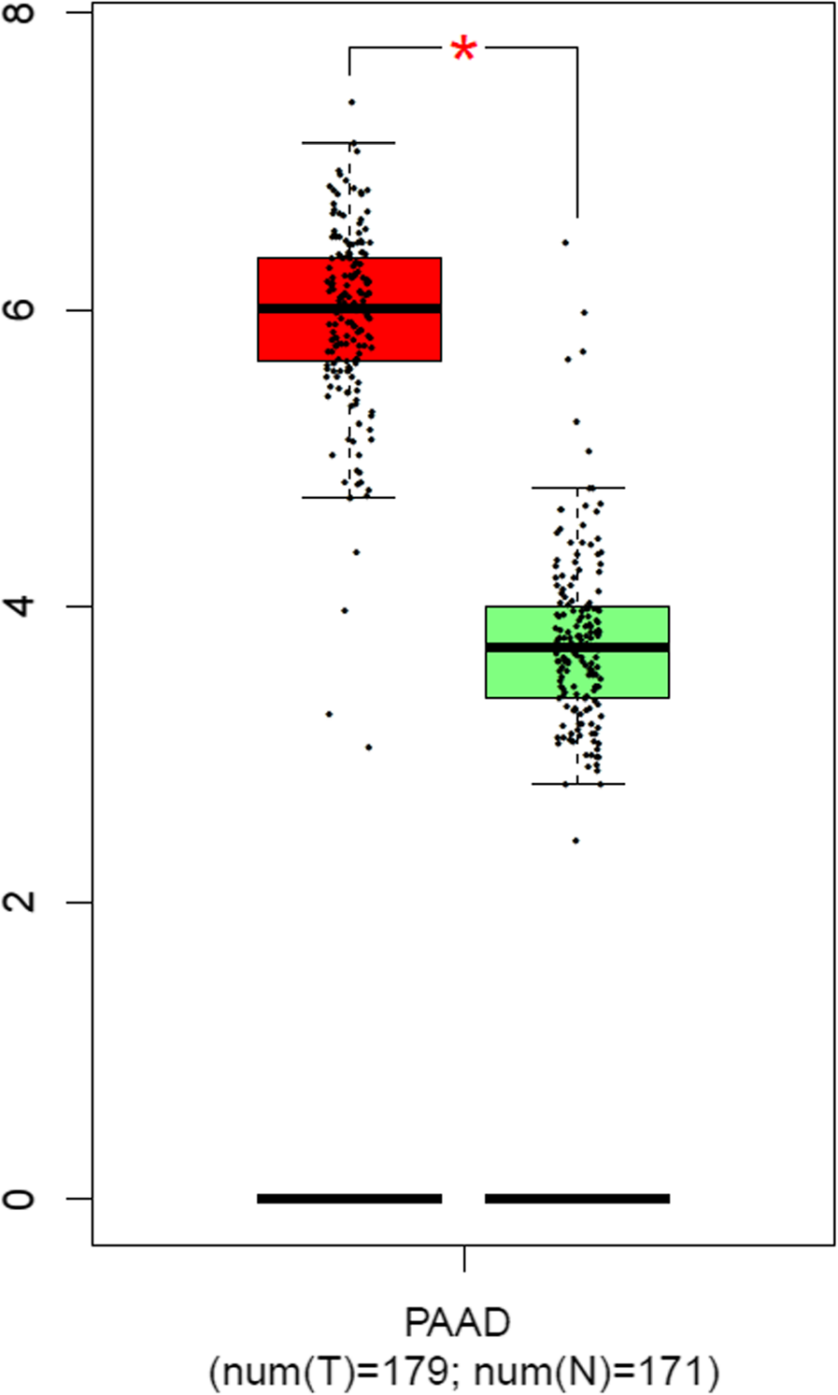
Expression level of YWHAG in pancreatic cancer and normal tissue in the TCGA database

**Fig. 8:**
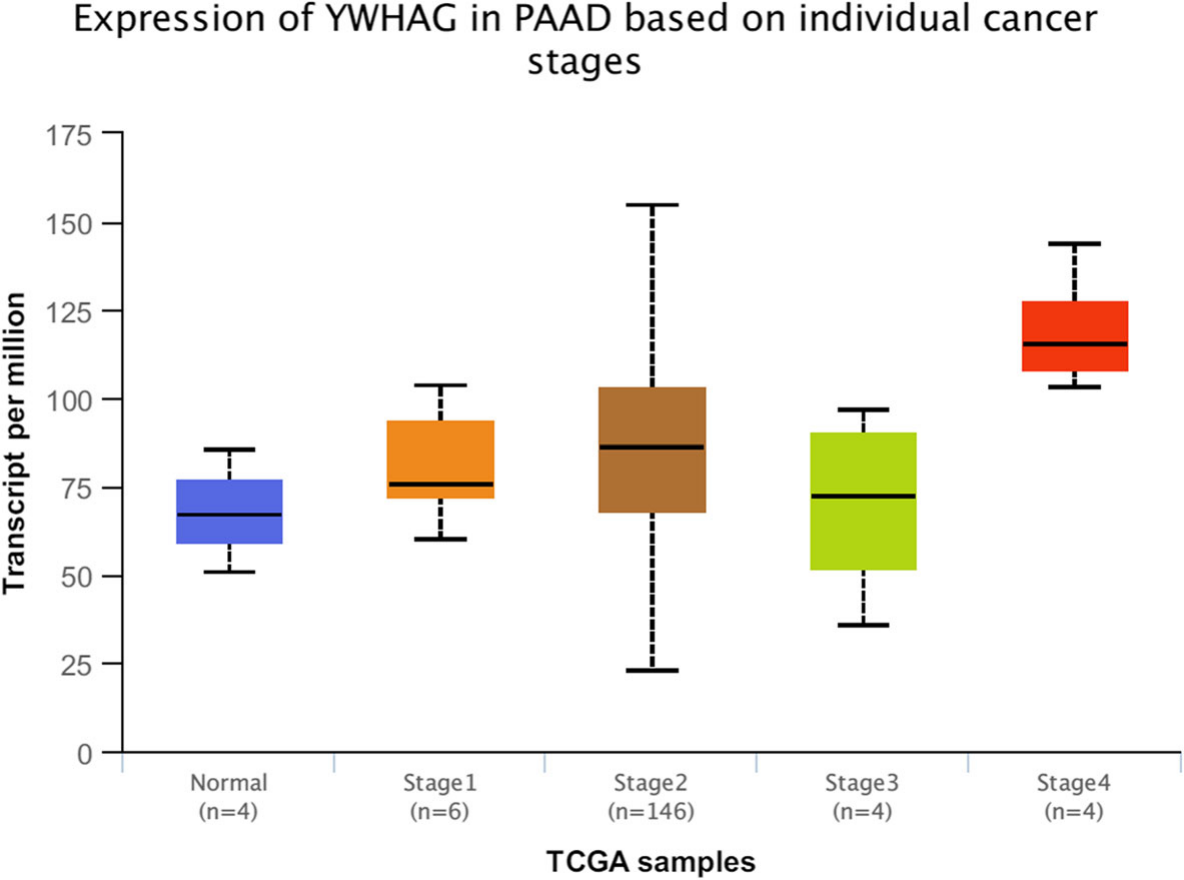
Expression level of YWHAG in different individual pancreatic cancer stages in the TCGA database

**Fig. 9:**
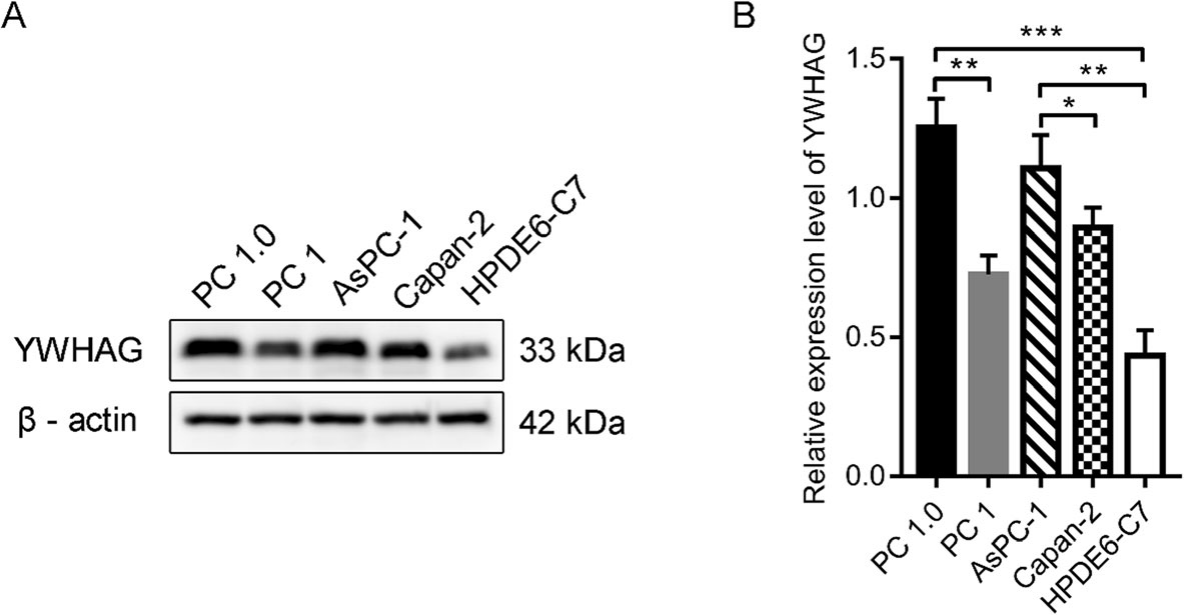
YWHAG protein levels were detected using western blot analysis. **a**, Western blot validation of YWHAG from cells lines. **b**, Quantitative analysis of YWHAG is shown. *P < 0.05,**P < 0.01, ***P < 0.001

**Fig. 10:**
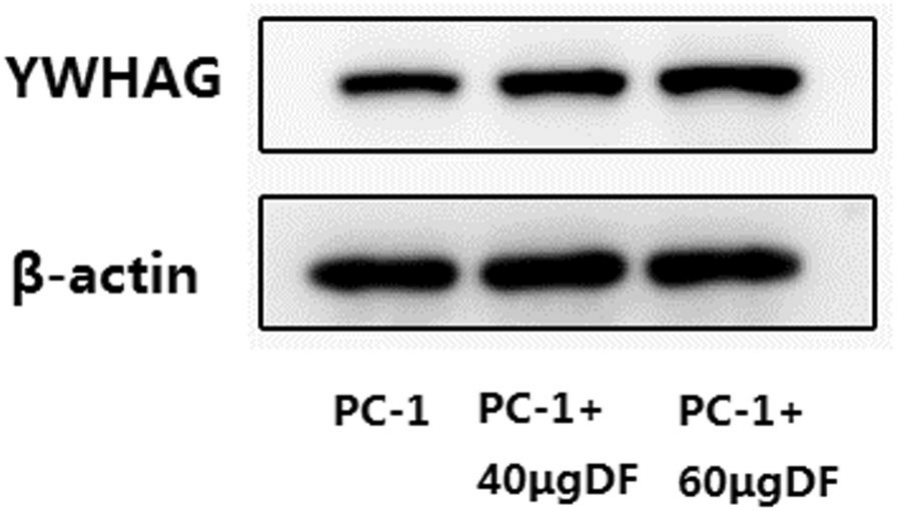
The expression level of YWHAG in PC-1 cells increased with the increase of cocultured PC-1.0-derived DF. **P < 0.01

Through online functional annotation cluster analysis with DAVID software, the secretory signal cluster was found (enrichment score: 1.91, Table 2). To further analyze the interaction between the identified proteins, we used the STRING database to retrieve the above six proteins (Fig. 5). We used online software to perform clinical correlation analysis of these six proteins. The results showed that YWHAG could be used as a prognostic biomarker for pancreatic cancer (Fig. 6). We used the Cancer Genome Atlas (TCGA) data visualization web tool GEPIA to analyze the expression of YWHAG in normal and cancer tissues. The results showed higher expression levels of YWHAG in pancreatic cancer than in normal tissue in the TCGA cohort (*P* < 0.01) (Fig. 7). Validation of the YWHAG expression level in different cancer stages from TCGA data showed higher expression levels of YWHAG in the advanced stage than in the early stage (Fig. 8). The Western blot results showed that YWHAG was highly expressed in the highly invasive PC-1.0 cell line (Fig. 9). The expression level of YWHAG in PC-1 cells increased with the increase of cocultured PC-1.0-derived DF (Fig. 10).

**Table 2:** DAVID Cluster Analysis

Enrichment Score: 1.9097618864692167		
Category	Term	*P*-Value
UP_KEYWORDS	Secreted	3.83E-04
UP_SEQ_FEATURE	disulfide bond	0.001971
UP_SEQ_FEATURE	signal peptide	0.003347
UP_KEYWORDS	Disulfide bond	0.003354
GOTERM_CC_DIRECT	GO:0005615~extracellular space	0.003597
GOTERM_CC_DIRECT	GO:0005576~extracellular region	0.006005
UP_KEYWORDS	Signal	0.006989

## Discussion

With the development of mass spectrometry technology, a large number of secreted proteins have been identified. These newly discovered proteins have been shown to be tumor markers [16]. When tumor cells secrete proteins into the extracellular environment, some of these proteins can change the tumor microenvironment and promote tumor growth [17]. In our previous work, we found that the supernatant of highly invasive PC-1.0 cells contained DFs. DFs can induce morphologic changes in and increase the invasive ability of low-invasive PC-1 cells. DFs play an important role in understanding the molecular mechanism of invasion and metastasis of cancer cells. In this study, we identified DFs using a mass spectrometry-based proteomic approach.

In this study, two different pretreatment methods were used to isolate samples by combined size exclusion chromatography (SEC) and to preliminarily analyze the secreted proteomes of pancreatic cancer cell lines. The results showed 1496 identified proteins in the serum-free medium group and 230 in the serum-contained medium group. In the serum-free experimental group, we found that the short-term serum starvation process can lead to changes in the secretory status. Therefore, the secretory status in serum-containing culture medium is more similar to the real secretory status and is thus more meaningful for later clinical verification and application. In the serum-containing medium group, we used SEC to isolate and enrich the secreted proteins in the supernatant. Although there was no quantitative data, the secreted proteins were also qualitatively identified through data mining and analysis. Six proteins were identified, namely, MMP12, MMP10, LAMA5, YWHAG, CPN1 and THPH2. Among these six proteins, MMP12 and MMP10 are members of the matrix metalloproteinase family, and have been proven to be closely related to pancreatic cancer [18, 19]. LAMA5 is an important component of the extracellular matrix, which can regulate cell adhesion and promote cancer cell metastasis [20]. CPN1 can prevent the accumulation of polypeptides and regulate the secretory hormone level [21]. Baine MJ et al. reported that the level of coagulation factor V was found to be significantly different in an analysis of peripheral monocytes from pancreatic cancer patients and might be related to tumor stage [22]. YWHAG is a member of a highly conserved family of proteins that participates in many intracellular signal transduction processes and plays an important role in cell survival and proliferation [23–26]. However, the role of YWHAG in pancreatic cancer progression is still unclear. In our previous intracellular proteomic study, YWHAG was also the key gene in the PPI network [9]. The expression of YWHAG was found to be closely related to pancreatic cancer stage via TCGA database analysis. Furthermore, survival analysis suggested that patients with higher expression levels of YWHAG showed a worse overall survival rate than those with lower YWHAG expression.

## Conclusion

Given the above findings, several alternative proteins were identified as pancreatic cancer dissociation factors via a pair of homologous pancreatic cancer cell lines with different metastatic abilities. These results provided us with more comprehensive information on pancreatic cancer invasion and metastasis. YWHAG is suggested as a potential prognostic biomarker and a sensitive therapeutic target for pancreatic cancer invasion and metastasis.

## Supplementary information

**Additional file 1.** Results of Mass Spectrometry in the serum-free group.

**Additional file 2.** Results of mass spectrometry in the serum-containing group.

**Additional file 3.** Hamster secreted protein Database.

## Data Availability

All data generated or analyzed during this study are included in this published article and its supplementary information files.
